# Daniel Bennett Saint John Roosa

**Published:** 1908-04

**Authors:** 


**A Monthly Review of Medicine and Surgery.**

*EDITOR:*

WILLIAM WARREN POTTER, M. D.

All communications, whether of a literary or business nature, books for review and exchanges, should be addressed to the editor 238 DELAWARE AVE., BUFFALO, N. Y.

VOL. LXIII. APRIL, 1908. No. 9

### Daniel Bennett Saint John Roosa

BORN APRIL 4, 1838, DIED MARCH 8, 1908.

NOT recently has death in such a marked manner invaded the ranks of the medical profession. Professor Roosa's conspicuous service was in organising a medical school for post-graduate work, the New York Post-Graduate Medical School and Hospital, the first institution of its kind, whose president he has been since its foundation in 1883, as well as its professor of diseases of the eye and ear.

Dr. Roosa died suddenly March 8, 1908, from heart disease at his home, No. 20 East 30th street, New York City, aged 69 years. He had seemed in his usual good health when he first arose, but just after he returned to his room from his morning bath he fell to the floor unconscious, and died in a few minutes.

Daniel Bennett Saint John Roosa was born in Bethel, N. Y., on April 4, 1838, and was the son of Charles B. Roosa and Amelie E. Foster Roosa. His early education was received at the district school of his native village, and at the academies of Monticello, N. Y., and Honesdale, Penn. In 1856 he entered Yale, but he was obliged to leave college, on account of ill health, in a few months. His studies were continued under a private tutor until the fall of 1857, when he entered the medical department of the University of the City of New York, from which he was graduated in 1860. He served as interne in the New York Hospital until April, 1861, when he enlisted as assistant surgeon to the Fifth New York Volunteers. Dr. Roosa served through the three months' term of enlistment of this regiment, and, returning to New York, completed his service in the New York Hospital as house surgeon. He then spent a year in Europe in study of the ophthalmic clinics in Berlin and Vienna. In June of 1863 he volunteered with the 12th Regiment, N. G. S. N. Y., and was sent to Pennsylvania, only a short time before the battle of Gettysburg.

On the completion of his term of service in the army he engaged in private practice in New York City, chiefly in ophthalmology and otology. From 1864 to 1882 he was professor of diseases of the eye and ear at the Medical School of New York University, and from 1875 to 1880 he held a similar place at the University of Vermont. In 1879 he was made president of the Medical Society of the State of New York, and in 1876 of the International Otological Society. He was also president of the American Otological Society, and honorary vice-president of the Ophthalmological Society, meeting in Edinburgh in 1894. He was president of the New York Academy of Medicine in 1893 and 1894, and was one of the founders and surgeons of the Manhattan Eye and Ear Hospital.

As a president of the Medical Society of the State of New York, Dr. Roosa distinguished his term of service by an address of unusual merit and by an administration that was unusually progressive in character. He was gifted as an orator, a man of graceful presence and a citizen of patriotic impulses. He was one of the leaders in the movement which led to the abolition of the old code of ethics in the state of New York, and was always particularly prominent in securing proper legislation on matters relative to the profession of medicine.

In addition to a number of works of his own on the eye and the ear, Dr. Roosa was a frequent contributor to medical journals and translated several medical works by Germans.

Two honorary degrees were conferred upon him, the degree of M. A. by Yale and of LL. D. by the University of Vermont. He was twice married, his first wife having been the daughter of Stephen M. Blake of New York, who died in 1878. His second wife, who was Mrs. Sarah E. Howe, the daughter of Eder V. Haughwout, survives him.

